# The role of locus coeruleus nucleus TRPV1 receptors in the development and expression of morphine dependence

**DOI:** 10.22038/ijbms.2019.35055.8338

**Published:** 2019-10

**Authors:** Iman Fatemi, Zahra Hadadianpour, Farangis Fatehi, Ali Shamsizadeh, Jalal Hasanshahi, Mitra Abbasifard, Ayat Kaeidi

**Affiliations:** 1 Research Center of Tropical and Infectious Diseases, Kerman University of Medical Sciences, Kerman, Iran; 2 Physiology-pharmacology Research Center, Research Institute of Basic Medical Sciences, Rafsanjan University of Medical Sciences, Rafsanjan, Iran; 3 Department of Physiology and Pharmacology, Rafsanjan University of Medical Sciences, Rafsanjan, Iran; 4 Department of Internal Medicine, Ali-Ebn Abitaleb Hospital, Rafsanjan University of Medical Sciences, Rafsanjan, Iran

**Keywords:** Dependence, Locus coeruleus, Morphine, Rat, TRPV1

## Abstract

**Objective(s):**

This study investigated the role of locus coeruleus (LC) nucleus TRPV1 receptors (TRPV1r) in the expression and development of morphine physical dependence by intra-LC administration of AMG9810 (selective TRPV1r antagonist) in male Wistar rats.

**Materials and Methods:**

For assessing the development of morphine dependence, AMG9810 (0.03 and 0.3 mM in 10% DMSO, 0.2 µl; intra-LC microinjection) was administered before each morphine administration for seven continues days (once daily; 6, 16, 26, 36, 46, 56, and 66 mg/kg; sc). Furthermore, for evaluating the expression of morphine dependence, a single dose of AMG9810 (0.03 and 0.3 mM in 10% DMSO, 0.2 µl; intra-LC microinjection) was administered to morphine-dependent rats on day 8 of the experiment.

**Results:**

Obtained data demonstrated that co-administration of TRPV1r antagonist with morphine reduced the development of morphine withdrawal syndrome somatic signs induced by naloxone. Moreover, single intra-LC administration of TRPV1r antagonist on the final day of the examination period significantly decreased the expression of some signs of morphine withdrawal in rats.

**Conclusion:**

The results showed that LC TRPV1r might be participating in the expression and development of morphine dependence.

## Introduction

Opioid drugs like morphine are the strongest analgesic drugs that are widely used for the relief of acute and chronic pain (1, 2). Chronic morphine consumption leads to the development of physical dependence (3, 4). If the opiate consumption is ceased abruptly, the withdrawal sings occurs, including chronic fatigue, leg and back pain, sleep disturbances, mood disorders, and restlessness (5). The underlying mechanisms that cause this process are still poorly understood (6). For many years, the morphine dependence conditions can be controlled or attenuated by some medications such as clonidine and methadone (7, 8). Opioid dependence is a multifaceted phenomenon that includes several mechanisms, including free radicals (reactive nitrogen and oxygen species) (9), down-regulation of opioid receptors (10), inflammation of the central nervous system (7) and activations of glutamate receptor NMDA (N-methyl-D-aspartate) (11). The locus coeruleus (LC) nucleus is one of the main brain organizations that is involved in the opiate withdrawal syndrome expression (12). Also, LC nucleus is one of the highly sensitive areas for induction of opiate withdrawal somatic signs (13). Moreover, blocking LC nucleus neuronal activity decreases several signs of opioid withdrawal in the animals (14). Furthermore, electrical lesions of the LC nucleus reduce the opiate withdrawal signs (15). 

TRPV1 (transient receptor potential vanilloid-1) receptors that belong to Transient receptor potential (TRP) family, which is a non-selective cation channel. These cationic channels have been confirmed as a pain target by capsaicin, low pH and, noxious stimuli. The TRPV1 receptors have a critical role in the initiation of the inflammatory responses (16, 17). The role of the central nervous system (CNS) TRPV1 receptors is not well known. These receptors contribute to the perception of pain, body temperature adjustment, control of locomotion, and anxiety (18, 19). TRPV1 receptors are expressed in different brain areas, including the locus coeruleus, cerebral cortex, cerebellum, hypothalamus, hippocampus, olfactory bulb, periaqueductal gray, striatum, amygdala, and thalamus (20). It was shown that morphine decreases the TRPV1 receptors expression in the amygdala (21). Moreover, opioid withdrawal increases the activity of TRPV1 receptors (22). 

So, as a result of the documents mentioned above, the current study was proposed to investigate the probable LC TRPV1 receptors role in the expression and development of morphine-induced dependence in rats.

## Materials and Methods

***Animals***

Sixty-four male Wistar rats (weight 275 ± 25 g) were used in this investigation. Rats were obtained from the animal house of Rafsanjan University of Medical Sciences. The animals were housed in groups (4 rats per plastic cage) and kept in a 12 hr light/dark cycle (lights on 08:00 to 20:00) with free access to food and water and maintained at constant temperature (23±2.0 °C). All experimental procedures were performed according to the guidelines for the care and use of laboratory animals in Rafsanjan University of Medical Sciences based on the European Communities Council Directive 24 November 1986 (86/609/EEC). Each rat was used only once in this study, and each group consisted of 8 animals. All behavioral experiments were carried out at the same time of the day. 

***Experimental groups***

The animals were randomly separated into eight experimental groups (n=8 for each experimental group): 

1) Saline: saline-treated rats (with bilateral LC cannula implantation) for seven days, which received naloxone (3 mg/kg, IP) to find out if there is any withdrawal.

2) Morphine: morphine-treated rats (with bilateral LC cannula implantation) for seven days, which received naloxone (3 mg/kg, IP) to evaluate morphine withdrawal signs.

3 and 4) Morphine+AMG9810: AMG9810 (0.03 and 0.3 mM, 0.2 µl) treated rats through bilateral LC cannula before each morphine administration for seven days (for assessment of the AMG9810 effect on the development of morphine dependence). 

5) Morphine+AMG9810 vehicle: AMG9810 vehicle (10% DMSO, 0.2 µl) treated rats through bilateral LC cannula before each morphine administration for seven days (for assessment of the AMG9810 vehicle effect on the development of morphine dependence).

6 and 7) Morphine+AMG9810: Morphine treated rats (for seven days) that received a single dose of AMG9810 (0.03 and 0.3 mM, 0.2 µl) through bilateral LC cannula at day 8 (for assessment of the AMG9810 effect on the expression of morphine dependence). 

8) Morphine+AMG9810 vehicle: morphine-treated rats (for seven days) that received AMG9810 vehicle (10% DMSO, 0.2 µl) through bilateral LC cannula at day 8 (for assessment of the AMG9810 vehicle effect on the expression of morphine dependence).

**Figure 1:**
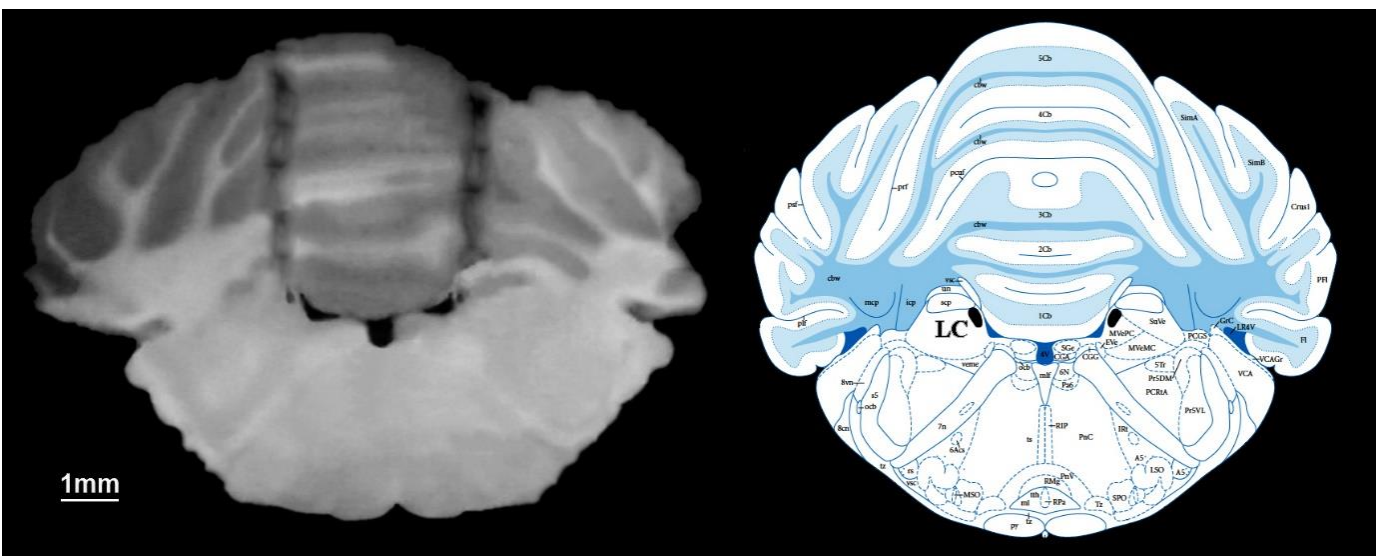
Cannula placements for bilateral intra-LC injections of AMG9810 or vehicle. The right panel shows a schematic representation of the rat brain section taken from the atlas of Paxinos and Watson (2005)(23). The left panel shows a photomicrograph of coronal brain section indicating the injection cannula tracks terminating into the right and left lateral LC nucleus

**Figure 2:**
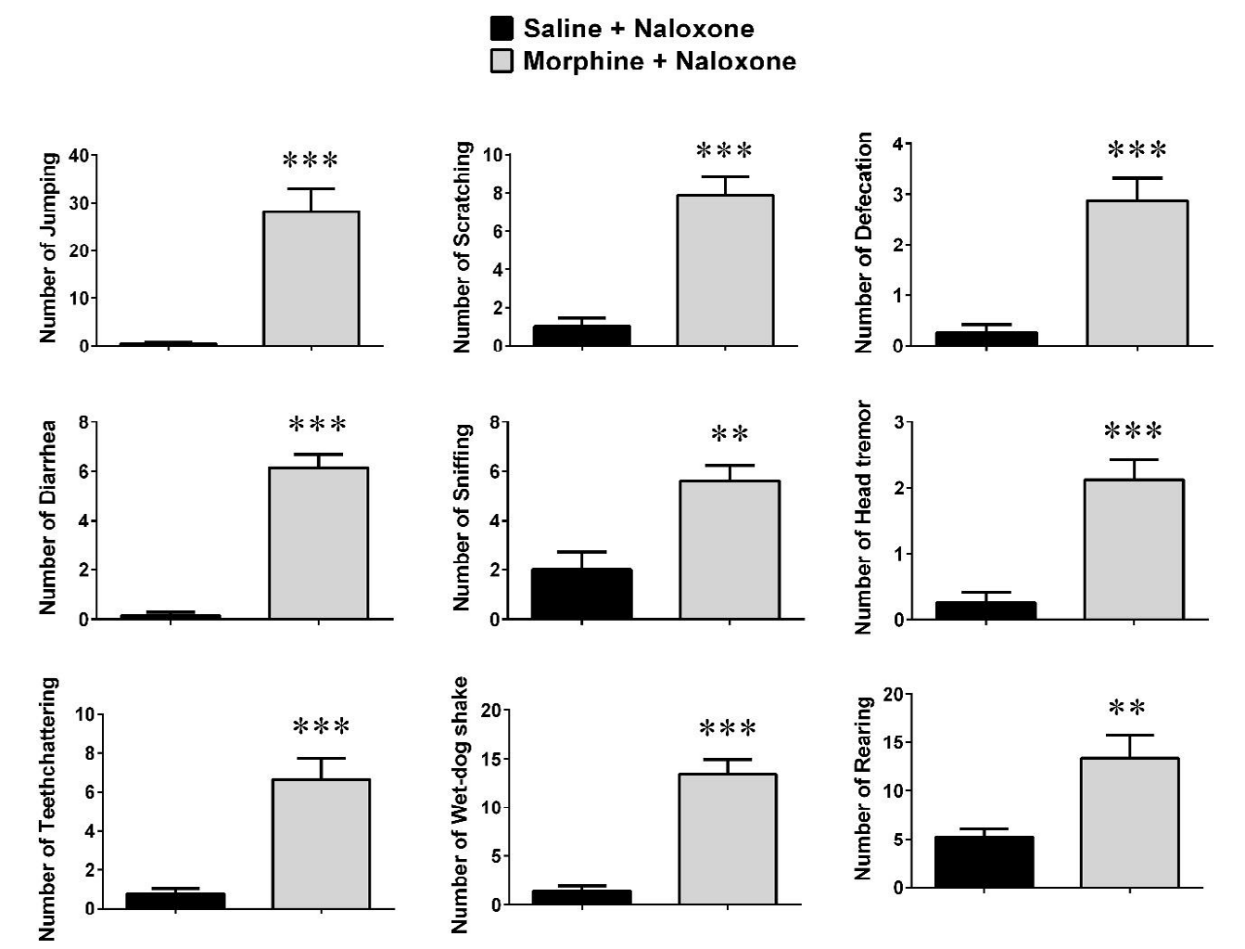
Naloxone-induced withdrawal sings in the morphine-dependent rats. Values are means±SEM (n=8). Morphine dependent rats (Morphine + Naloxone) are compared with the saline-treated rats (Saline + Naloxone group, as non-morphine dependent animals)

** *P*<0.01 and *** *P*<0.001 compared to morphine + naloxone group

**Figure 3:**
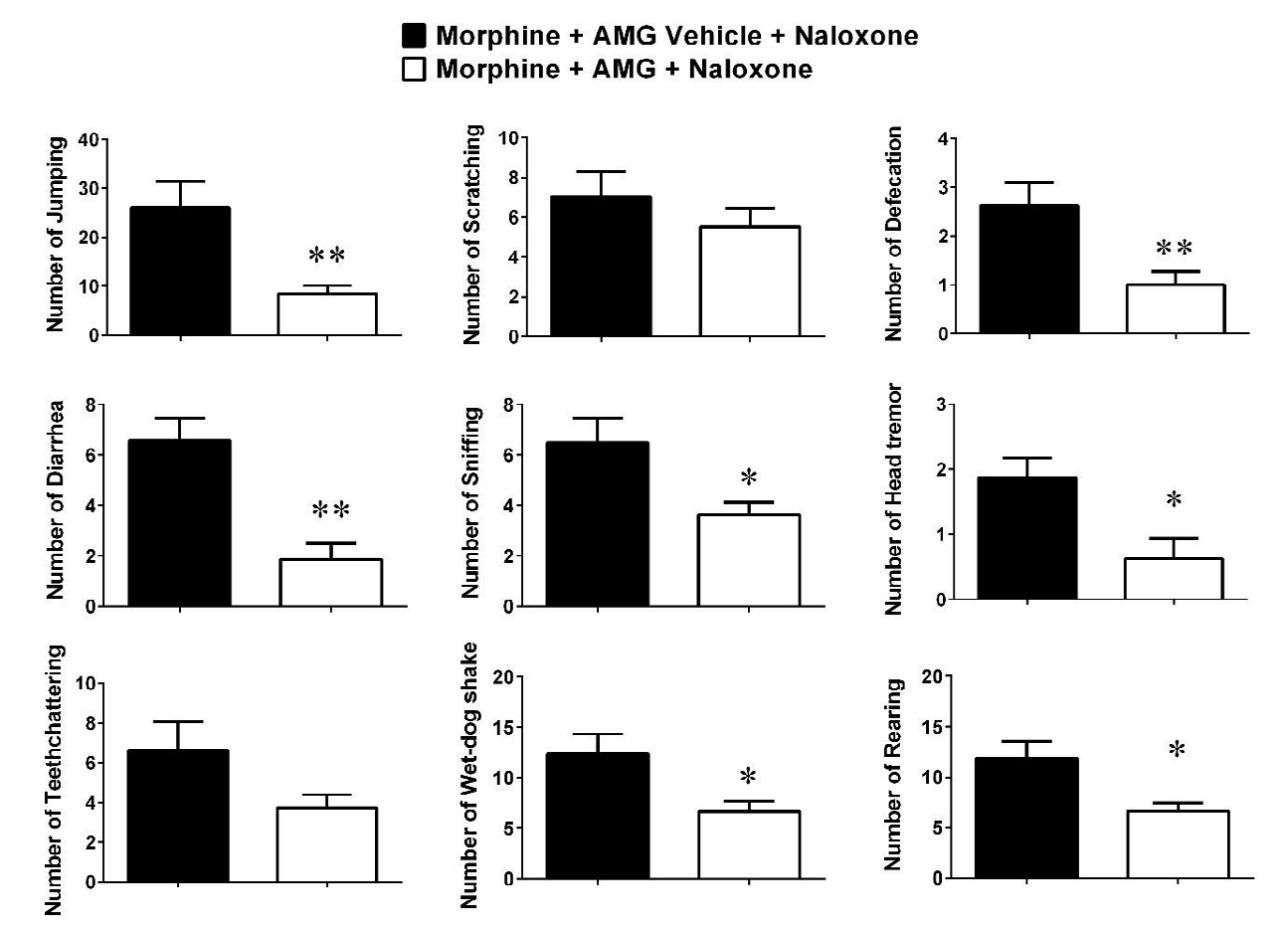
Effect of intra-LC microinjection of AMG9810 on the development of morphine dependence. Values are means±SEM (n=8). Morphine dependent rats receiving 0.3 mM LC AMG9810 (Morphine + AMG + Naloxone group) were compared with the ones pretreated with AMG9810 vehicle (Morphine+AMG Vehicle+Naloxone). * *P*<0.05 and ** *P*<0.01

**Figure 4:**
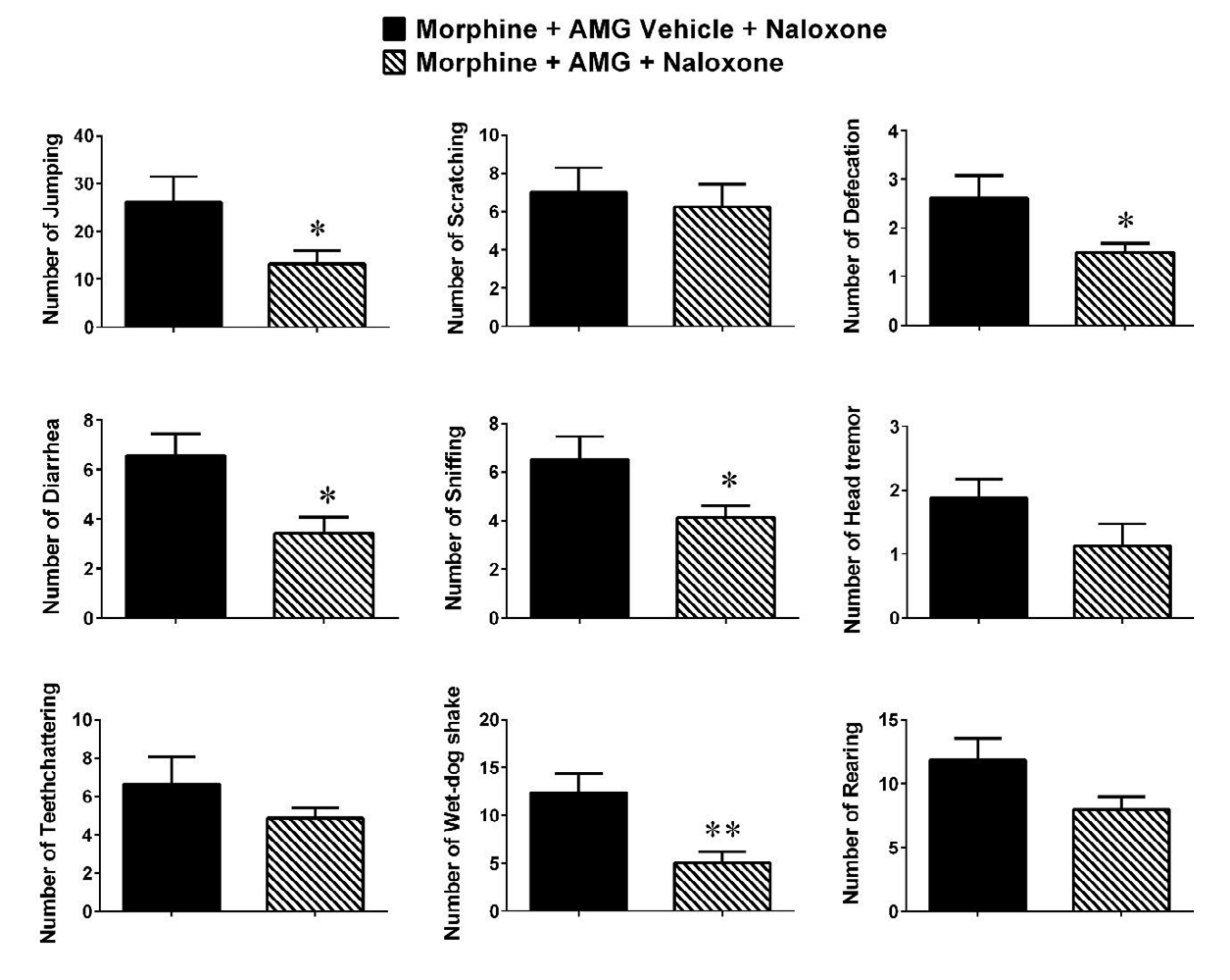
Effect of intra-LC microinjection of AMG9810 on the expression of morphine dependence. Values are means ± SEM (n = 8). The morphine-dependent rats receiving a single dose of AMG9810 on day 8 (Morphine + AMG + Naloxone group) were compared with the ones pretreated with AMG9810 vehicle (Morphine + AMG Vehicle + Naloxone). * *P*<0.05 and ** *P*<0.01

***Drugs***

AMG9810 (selective TRPV1 antagonist) (Tocris, Bristol, UK), Naloxone hydrochloride (Tolidaru, Iran), morphine sulfate (Temad, Iran), xylazine (Alfasan, The Netherlands), and ketamine (Alfasan, The Netherlands) were used in this investigation. Naloxone hydrochloride and morphine sulfate were dissolved in 2 ml normal saline (0.9%) to prepare fresh solution before each intraperitoneal injection. 

***Surgical procedure***

The rats were anesthetized by administration of ketamine 10% (100 mg/kg; IP) and xylazine 2% (10 mg/kg; IP). Then, the rats were mounted in a stereotaxic apparatus. After the skull surface was exposed, we inserted two guide cannula (13 mm long, 23 ga stainless-steel needle) with a precisely fitted stylet of equal length bilaterally 1 mm above the microinjection site in the LC nucleus (-9.8 mm caudal to bregma, 1.3 mm from the medial suture, and -7.2 mm below the surface of skull) (23). The guide cannula was fixed to the surface of the skull using two small stainless-steel screws that were firmly fixed to dental acrylic cement. Figure 1 shows cannula placement for the injections of AMG9810 or vehicle into the right ventricle LC nucleus.

***Induction of morphine dependence***

Morphine treatment was started after surgery recovery time (10 days) and continued for 7 consecutive days (once daily; 6, 16, 26, 36, 46, 56, and 66 mg/kg; sc). Non-morphine dependent rats received the saline at an equal volume. Injections of drugs were done at a similar time during the entire experiment period. Naloxone (3 mg/kg, IP) was used for induction of morphine withdrawal syndrome 2 hr after the last dose of morphine administration on the 8th day (12, 24).

***Microinjection of AMG9810***

AMG9810 was dissolved in sterile 10% dimethyl sulfoxide (DMSO) and kept frozen (-20 ^°^C). AMG9810 microinjection was done after guide cannula stylet dismounting and lowering an injecting cannula down (14 mm, 30 ga stainless-steel needle) in a way that extended 1 mm outside the tip of guide cannula and was attached to a 1 µl Hamilton syringe by polyethylene tubing. Then, 0.2 µl of AMG9810 or its vehicle (DMSO 10%) were injected within 1 min. To avoid the backflow of the injected drugs, the injecting cannula was carefully removed 1 min after microinjection and the stylet was replaced gently. 

***Monitoring the morphine withdrawal symptoms***

Morphine withdrawal syndrome was induced by IP injection of 3 mg/kg of the naloxone hydrochloride 2 hr after the last administration of morphine. Subsequently, nine different somatic signs of morphine withdrawal, including jumping, diarrhea, writhing, digging, sniffing, rearing, teeth chattering, scratching, and defecation were exhibited. We monitored the mentioned withdrawal signs in a test chamber with a transparent Plexiglas wall (35 cm long, 35 cm wide, 45 cm high) through a 30 min time course. The same observer who was blind to the groups did all experiments. The number of morphine withdrawal somatic signs was recorded during the whole experiment time.

***Assessment of the AMG9810 on morphine dependence development***

For assessment of TRPV1 effects on the morphine dependence development, two doses of AMG9810 (0.03 and 0.3 mM in 0.2 µl volume) or its vehicle (0.2 µl DMSO 10%) were administered through bilateral LC cannula immediately before every morphine administration for seven days. 

***Assessment of the AMG9810 morphine dependence expression***

For assessing the TRPV1 effects on the morphine dependence expression, animals that had received only morphine for seven consecutive days were used. The AMG9810 (0.03 and 0.3 mM in 0.2 µl volume) or its vehicle (0.2 µl DMSO 10%) were administered through bilateral LC cannula only on the 8th day, 45 min prior to morphine administration. 

***Histological verification***

After the end of behavioral assessments, we verified the injection sites by administrating the pontamine sky blue 2% (0.1 µl) to the injection site of animals. Then, the animals’ brains were carefully removed from the skull and fixed in formalin solution (10%) for 24 hr. Serial coronal sections of the rat brains were performed, and the site of cannula tips was verified using Paxinos and Watson rat brain atlas. 

***Statistical analysis***

Analysis of the data was done via GraphPad Prism version 6.01 for Windows (GraphPad Software, USA). Statistical differences among the groups were tested using Student’s t-test. If *P*<0.05, the differences among means were statistically considered significant. The results are presented as mean±SEM.

## Results

Following naloxone administration (3 mg/kg, IP), saline-treated rats demonstrated no withdrawal signs, whereas morphine-dependent animals exhibited signs of withdrawal, including jumping, scratching, defecation, diarrhea, sniffing, head tremor, wet-dog shake, teeth chattering, and rearing (Figure 2). All of the mentioned withdrawal signs were statistically significant compared to saline-administrated rats, confirming the development of morphine dependence. 

Administration of AMG9810 vehicle (0.2 µl DMSO 10%) into the LC, made no significant difference in the development of morphine dependence. However, the morphine-dependent animals that received the AMG9810 (0.3 mM, for seven days) exhibited a significant decrease in many signs of naloxone-induced withdrawal symptoms except scratching and teeth chattering (Figure 3). It is notable that, AMG9810 administration at the dose of 0.03 mM did not induce significant effects on measured indices; so, those data are not shown. 

To assess whether AMG9810 could inhibit the naloxone-induced morphine withdrawal expression in the established tolerance, we treated the rats with AMG9810 (0.3 mM 0.2 µl volume) or its vehicle (0.2 µl DMSO 10%) in morphine-treated animals 45 min prior to the last dose of morphine (10 mg/kg, sc) on day 8. Naloxone (3 mg/kg; IP) was administrated 2 hr after the last dose of morphine injection, and rats were monitored for withdrawal signs during 30 min. Data indicated that AMG could prevent the expression of several withdrawal somatic signs such as jumping, diarrhea, sniffing, wet-dog shake, and defecation (Figure 4). The results showed that AMG at the dose of 0.03 mM did not induce significant effects on measured indices; so, those data are not shown.

## Discussion

The results of our study indicated that administration of AMG9810 (selective TRPV1 receptor antagonist) in LC before each morphine administration reduces the development of morphine dependence in rats. Our findings also demonstrated that blocking TRPV1 receptors in LC significantly reduced the expression of several somatic signs of naloxone-induced morphine withdrawal, such as jumping, defecation, wet-dog shake, head tremor, diarrhea, teeth chattering, and scratching. Additionally, these results indicate that microinjection of AMG9810 into the LC nucleus had no considerable effects on scratching and teeth chattering in morphine dependence development. These phenomena may be due to this fact that TRPV1 receptors located in the LC nucleus might participate in the expression and development of some physical signs of morphine withdrawal. 

Our obtained results are in line with previous investigations. It was shown that blocking the nucleus accumbens (NAc) TRPV1, attenuates the morphine conditioned place preference without affecting normal activity (25). In another investigation, it was demonstrated that injection of TRPV1 antagonist in NAc decreases the phospho-p38 mitogen-activated protein kinase (MAPK), adenylyl cyclase type 1 and nuclear factor-kappa B (NF-κB) protein expression levels in the NAc of morphine-dependent mice (26). Furthermore, blocking the TRPV1 significantly decreases morphine self-administration, morphine-induced c-fos expression in the NAc, and anxiolytic-like effect during the period of morphine abstinence (27).

The exact cellular mechanisms through which block of TRPV1 receptors attenuates symptoms of withdrawal are not clearly understood. Nevertheless, alterations in the glutamate receptors activity, which modulates the oxidative stress and inhibits the inflammatory processes might be involved in this trend. It has been shown that chronic morphine administration may lead to induced inflammation in the brain (28). Previous findings have revealed that TRPV1 receptors can be activated by inflammatory processes, and activation of TRPV1 could increase the release of cytokines/chemokines (29, 30). Emerging studies have shown that inhibiting the TRPV1 receptors reduced the neurogenic inflammation (31, 32). So, there is the possibility that blocking TRPV1 receptors could attenuate the expression and development of morphine dependence via reducing morphine-induced inflammation. 

The glutamate and its receptors activation role in the development and expression of morphine dependence have been well established (33). It is well documented that morphine activates glutamate receptors and increases calcium ion inflow (11). It has been demonstrated that MK-801 could prevent morphine-induced dependence via blocking the NMDA receptor (34). Also, Kaeidi *et al*. showed blockage of glutamatergic inputs to LC can reduce the naloxone-induced LC neuronal hyperactivity in the brain slices of morphine-dependent rats (35). Furthermore, Nishio *et al*., indicated that reactive oxygen species (ROS) generation and oxidative stress increase the spontaneous glutamate release from presynaptic terminals through activation of the TRPV1 channel (36). Another study, showed that TRPV1 activation potentiates the release of glutamate from primary afferent nerve fibers excitatory terminals (37). TRPV1 activation was accompanied by elevation in intracellular Ca^2+^, and this effect was inhibited by capsazepine (TRPV1 receptor antagonist) (30). So, there is the possibility that these effects may be due to the inhibition of glutamate neurotransmitter and receptors.

Chronic morphine administration increased oxidative stress in the brain and spinal cord (38). Antioxidant compounds can mitigate the physical signs of morphine dependence (39). Moreover, activating TRPV1 increased cytosolic free Ca2+ and oxidative stress (40). On the other hand, TRPV1 channel inhibition is associated with improving antioxidant functions (41). Hence, it seems that blocking the TRPV1 receptor may reduce the expression and development of morphine dependence via the antioxidative mechanism.

## Conclusion

It seems that TRPV1 receptors in the LC nucleus have a critical task in the expression and development of morphine dependence. Our results propose that morphine treatment with TRPV1 antagonism combination might be helpful to protect against morphine physical withdrawal syndrome when the treatment is discontinued. Undoubtedly, additional studies are necessary to reveal the exact role of TRPV1 in the expression and development of morphine dependence.
